# The epidemiology of *Staphylococcus aureus* carriage in patients attending inner city sexually transmitted infections and community clinics in Calgary, Canada

**DOI:** 10.1371/journal.pone.0178557

**Published:** 2017-05-25

**Authors:** Alejandra Ugarte Torres, Angel Chu, Ron Read, Judy MacDonald, Daniel Gregson, Thomas Louie, Johanna Delongchamp, Linda Ward, Joann McClure, Kunyan Zhang, John Conly

**Affiliations:** 1 Division of Infectious Diseases, Department of Medicine, University of Calgary and Alberta Health Services, Calgary, Alberta, Canada; 2 Sexually Transmitted Infections Clinic, Alberta Health Services, Calgary, Alberta, Canada; 3 Department of Microbiology, Infectious Diseases and Immunology, Cumming School of Medicine, Calgary, Alberta, Canada; 4 Snyder Institute for Chronic Diseases, Cumming School of Medicine, University of Calgary and Alberta Health Services, Calgary, Alberta, Canada; 5 Department of Community Health Sciences, Cumming School of Medicine, University of Calgary, Calgary, Alberta, Canada; 6 Infection Prevention & Control, Alberta Health Services, Calgary, Alberta, Canada; 7 Department of Pathology and Laboratory Medicine, Cumming School of Medicine and Alberta Health Services, Calgary, Alberta, Canada; 8 O’Brien Institute for Public Health, Cumming School of Medicine and Alberta Health Services, University of Calgary, Calgary, Alberta, Canada; 9 Centre for Antimicrobial Resistance, Alberta Health Services and University of Calgary, Alberta, Canada; 10 Calgary Laboratory Services, Alberta Health Services, Calgary, Alberta, Canada; University of Queensland, AUSTRALIA

## Abstract

**Background:**

Although the nares represent the most common carriage site for traditional hospital-associated strains of *Staphylococcus aureus* (SA), the predominant site of carriage of SA in the community is less certain.

**Methods:**

We conducted a cross-sectional study in 285 patients attending sexually transmitted diseases and inner-city clinics to evaluate the prevalence, body site colonisation and risk factors associated with carriage of methicillin susceptible SA (MSSA). All isolates were characterized by pulsed field gel electrophoresis, staphylococcal cassette chromosome *mec*, staphylococcal protein A and multilocus sequence typing.

**Results:**

The prevalence of colonisation with SA was 57.5% (164/285); 162 (56.8%) participants were colonized with MSSA, and 4 (1.4%) with methicillin-resistant SA (MRSA), 2 of them were co-colonised with both MRSA and MSSA. The most common sites of colonisation were the throat (73.1%), nares (65.2%) and interdigital web spaces of the hand (21.3%). Three out of 4 MRSA isolates were USA300-MRSA strains. Twelve MSSA isolates were closely related to the USA300 CA-MRSA. We identified sexual behaviours such as having more than 6 heterosexual sexual partners in the last 6 months and trimming pubic hair to be independently associated with MSSA colonisation, and more specifically practicing oral sex as a risk factor for throat colonisation.

**Conclusion:**

There is a high prevalence of MSSA carriage in this population, with a low prevalence of MRSA. The throat was the most common site of carriage and sexual behaviours were found to be risk factors for MSSA colonisation. Close strain relatedness of MSSA and USA300-MRSA isolates suggests either gain or loss of the SCC*mec* element, respectively.

## Introduction

Community-acquired strains of methicillin-resistant *Staphylococcus aureus* (CA-MRSA) have been recognized over the last decade with increasing frequency among patients in ambulatory and hospital settings throughout Canada [1] and the United States. [2] CA-MRSA infections are defined by the lack of exposure to the hospital setting and phenotypically by the characterization of the SCC*mec* genotype, pulsed field electrophoresis pattern, multilocus sequence typing and the presence of Panton-Valentine leukocidin (PVL) toxin genes. [3] The dominant CA-MRSA clone in Canada has been USA-300 since it was first identified in 2004. [4] CA-MRSA has been associated with severe infections in vulnerable populations, often associated with illicit drug use, homelessness and incarceration. [5,6] The predominant site of carriage of CA-MRSA strains is unclear. In a US national population-based survey the prevalence of MRSA nasal colonisation was 1.5%, and less than 20% of them were USA300 isolates. [7] This relatively small proportion of nasal colonisation does not explain the increasing numbers of CA-MRSA infections [8], raising the possibility of extra-nasal body colonisation.

Other body sites of *Staphylococcus aureus* colonisation have been recognized including the genital area, as described by a recent study from a population of patients attending a sexually transmitted infections (STI) clinic in Baltimore. [9] This study found that the prevalence of genital colonisation with *Staphylococcus aureus* (SA) is high in the community (~ 50%) and nearly one fifth of colonised individuals would be missed if the genital area was not tested. Lee and colleges identified having a sexual partner with a skin infection as a risk factor for MRSA colonisation amongst HIV-positive patients [10] Similarly Diep and colleagues [11], on a population-based survey amongst men who have sex with men (MSM) in Boston and San Francisco, found a higher prevalence of CA-MRSA infections amongst MSM and up to 27% of them were infections in the genital area, suggesting close contact as risk factor for CA-MRSA infections. This observation is supported by the Cook et al’s [12] findings of CA-MRSA colonisation followed by infections in the pelvic area amongst 3 heterosexual couples. These findings suggest that close contact may play a role in the transmission of SA among couples, especially in the pelvic area.

We undertook a cross-sectional study of carriage of SA, both methicillin-resistant and methicillin-susceptible, in at-risk populations from STI and inner city clinics in Calgary, Alberta, Canada. The objectives of the study were to determine the prevalence of colonisation with SA, rates of carriage at different body sites and to identify risk factors associated with SA carriage.

## Methods

### Recruitment

Between February and November 2014, all individuals attending the STI clinic and an inner city medical clinic: Calgary Urban Project Society (CUPS) were recruited to participate. Inner city clinics provide care to underserved and minority groups in a densely populated central core area of the city. Individuals 18 years of age or older, with no previous hospitalization over last 4 weeks and no use of antimicrobials with anti- staphylococcal activity within the last 6 weeks were eligible to participate. The University of Calgary Conjoint Health Research Ethics Board approved this study.

### Sample size

A sample size of 141 individuals was estimated based on an expected colonisation rate with CA-MRSA USA300 of 8% [9], with a 90% power and 95% confidence (Stata 14, StataCorp^®^, College Station, TX).

### Data collection

After written informed consent was obtained, a 50-item questionnaire (S1 File) was administered by AUT or JC, which focused on demographics, medical, social and sexual behaviour. The relevant nominal information was anonymized and transferred to a protected purpose-built database within an Alberta Health Services password-protected, encrypted drive. Culture swabs (Copan Transystem^®^, Murrieta, CA) were collected from traditional sites: nares, throat, axilla, inguinal skin folds, and vagina, and from 3 novel sites: upper back, interdigital web spaces (IDWS) of hands and perineal-perianal area (“key swab”). The key swab was obtained applying the swab with a continuous movement starting at the upper margin of the perineum and moving downwards to the margins of the anal region and then around it, resembling the shape of a skeleton key.

### Laboratory methods

Samples were inoculated in 5 ml tryptic soy broth with 7% sodium chloride for 72 hrs at 37°C. Following incubation, broths were subcultured to mannitol salt agar plates and incubated 48 hr at 37°C. [13] Presumptive SA isolates were subcultured to blood agar and Denim Blue plates (48 hr, 37°C) for confirmation and to assess purity. [14] Any presumptive SA not readily identifiable by morphology and the above cultures were investigated further with a coagulase test (Becton Dickinson^®^ catalogue # 240826) and/or Staphylsoside Latex kit (Becton Dickinson^®^ catalogue #240952). MRSA were further confirmed by PCR assay for *nuc*, *femA* and *mecA* genes as previously described. [15] All isolates were typed by pulsed-field gel electrophoresis (PFGE) according to the Canadian standardized protocol. [16] The strains were tested for the presence of Panton Valentine Leukocidin (PVL) genes. [17] Any MRSA isolates were characterized by staphylococcal cassette chromosome *mec* (SCC*mec*) typing [18,19], and all isolates were characterized by staphylococcal protein A *(spa)* typing [20] and multilocus sequence typing (MLST). [21] Identification of strains matching the MRSA USA300 strain was based on the following: SCC*mec* type IVa, *spa* type t008, MLST type ST8 and identical PFGE pattern with standard USA300 control strain CA-MRSA-10.

### Data analysis

Participants with missing questionnaire or laboratory data were excluded from analysis. Univariate analysis was performed using Pearson’s chi-square or Fisher exact test for categorical data and independent sample t-test or Wilcoxon rank sum test as appropriate for continuous data to assess for possible independent risk factors associated with MSSA colonisation [22]. Subsequently, all variables that showed statistical and biological significance with a p <0.1 were included in logistic regression model analysis. Odds ratios and 95% confidence intervals were calculated with a level of statistical significance set at p<0.05. Stata14 (StataCorp^®^, College Station, Texas) was used for the statistical analysis.

## Results

**Fig 1:**
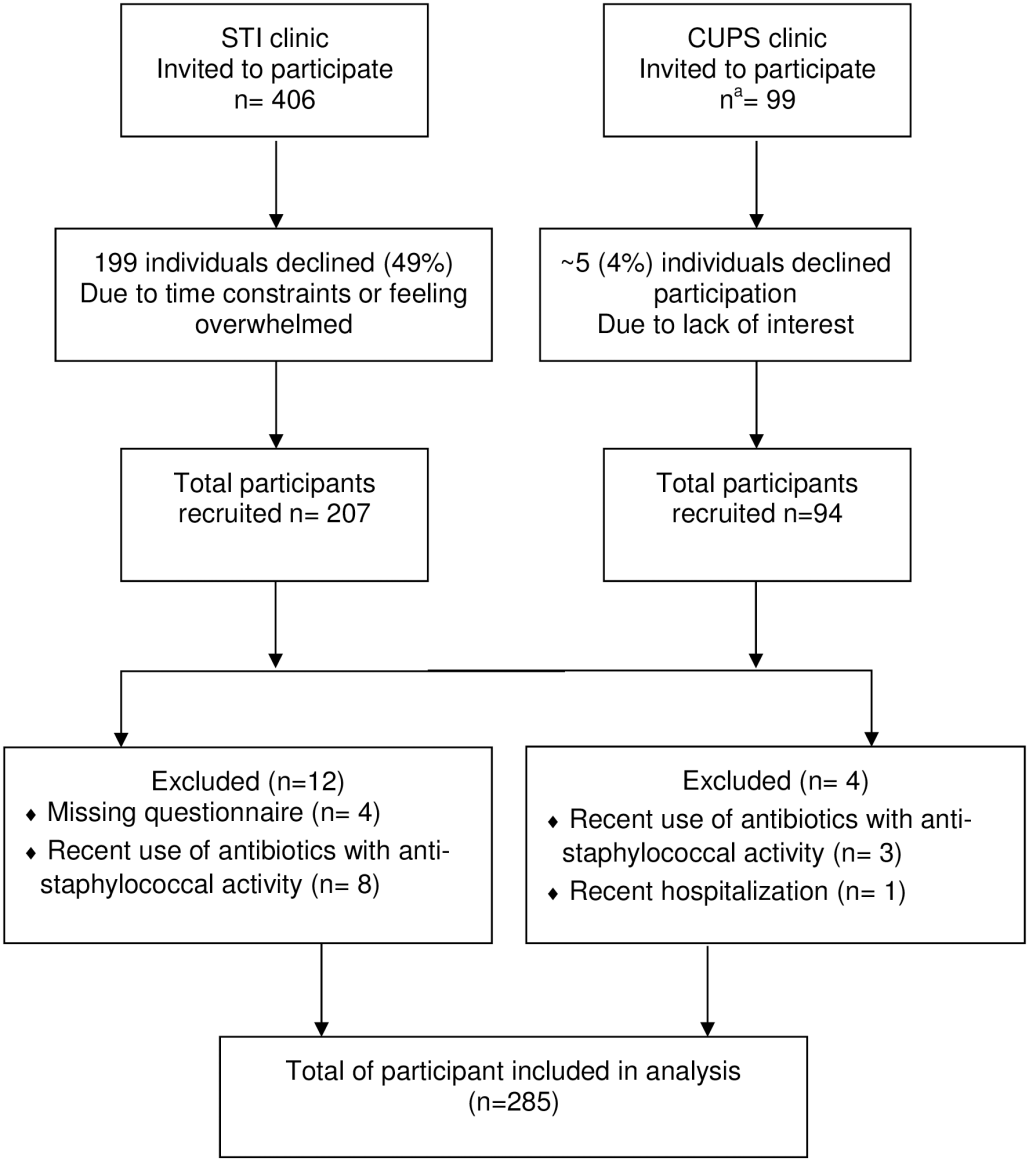
Flow diagram of study participants. ^a^Estimated number.

Overall, 301 participants were recruited and 285 included in the analysis (Fig 1), with 68% individuals from the STI clinic and 32% from CUPS. Participants were predominantly men (Table 1) and the mean overall age was 37.1 years. Participants recruited at CUPS vs. STI clinic were older, more likely to be of aboriginal ethnicity, unemployed, live in shelters, had a recent incarceration and to have used intravenous (IV) drugs. CUPS participants attended the clinic for reasons not related to an STI, including dental care, management of hepatitis C virus infection and prenatal care.

**Table 1:** Baseline characteristics of 285 study participants by site of recruitment.

Characteristic	Total n = 285	STI clinic n = 195	CUPS n = 90	*p*
	n %	n (%)	n (%)	
Age-mean (range)	37.1 (18–71)	33 (18–69)	46 (19–71)	<**0.01**
Male	186 (65.2)	131 (67.2)	55 (61.1)	0.31
Ethnicity:				
Caucasian	225 (78.9)	163 (83.5)	62 (68.9)	**<0.01**
Aboriginal	25 (8.7)	8 (4.1)	17 (18.9)	**<0.01**
Other	35 (12.2)	24 (12.3)	11 (12.2)	0.98
Unemployment	88 (30.8)	27 (13.8)	61 (67.8)	**<0.01**
Incarceration within the last 6 months	5 (1.75)	0 (0)	5 (5.6)	**<0.01**
Living in a Shelter	40 (14.0)	2 (1.0)	38 (42.2)	**<0.01**
Recreational illicit drug use	142 (49.8)	101 (51.8)	41 (45.6)	0.32
IV illicit drug use	11 (3.8)	3 (1.5)	8 (8.9)	**<0.01**
Self-reported ever being diagnosed:				
HIV infection	6 (2.1)	5 (2.6)	1 (1.1)	0.42
HCV infection	23 (8.1)	3 (1.5)	20 (22.2)	**<0.01**
Antibiotic use in the prior 6 months	84 (29.4)	54 (27.7)	30 (33.3)	0.33
Self-reported infected wounds^a^	16 (5.6)	12 (6.2)	4 (4.4)	0.56

^a^ In the last 6 months.

### Prevalence of colonisation

The overall SA colonisation rate was 57.5%, MSSA and MRSA carriage rates were 56.8% and 1.4% respectively. Two individuals had co-carriage of MRSA and MSSA. Three out of 4 MRSA colonised participants carried the USA300 strain, all MRSA carriers were recruited at CUPS. Overall, colonisation by MSSA was greater among STI clinic compared to CUPS participants (64.6% vs. 40%, p = <0.01). No SA infections were identified among participants.

### Site of colonisation with *S*. *aureus*

The most common site of SA colonisation was the throat, followed by the nares (Table 2). Overall, 18.3% participants were colonised at a pelvic site defined as any site of: groin, perineal-perianal or vaginal. Vaginal SA colonisation was found in 13.2%. Interestingly, the novel sites tested: upper back, IDWS and perineal-perianal revealed colonisation rates of 14%, 21.3% and 17.7% respectively. The rates of SA colonisation by body site were similar between the two recruiting sites, with only a trend towards higher rate of throat colonisation among the STI clinic (76.2%) vs. CUPS participants (61.1%), p = 0.07.

**Table 2:** Body site of colonisation with *Staphylococcus aureus* (SA), methicillin-sensitive *Staphylococcus aureus* (MSSA) and methicillin-resistant *Staphylococcus aureus* (MRSA) by site of recruitment.

Culture body site	SA positive			MSSA positive			MRSA positive^a^
	Total n = 164	STI clinic n = 126 (64.6%)	CUPS n = 38 (42.2%)	Total n = 162	STI clinic n = 126 (64.6%)	CUPS n = 36 (40%)	Total n = 4
	n %	n %	n %	n %	n %	n %	n %
Nares	107 (65.2)	80 (63.5)	27 (71.1)	104 (64.2)	80 (63.5)	24 (66.7)	3 (75)
Throat	120 (73.1)	96 (76.2)	24 (63.2)	118 (72.8)	96 (76.2)	22 (61.1)	2 (50)
Upper back	23 (14.0)	19 (15.1)	4 (10.5)	22 (13.6)	19 (15.1)	3 (8.3)	1 (25)
IDWS^b^	35 (21.3)	29 (23.0)	6 (15.8)	33 (20.4)	29 (23.0)	4 (11.1)	2 (50)
Axillary	19 (11.6)	18 (14.3)	1 (2.6)	19 (11.7)	18 (14.3)	1 (2.8)	-
Groin	30 (18.3)	23 (18.2)	7 (18.4)	29 (17.9)	23 (18.7)	6 (16.7)	1 (25)
Perineal-perianal	29 (17.7)	21 (16.7)	8 (21.1)	29 (17.9)	21 (17.1)	8 (22.9)	-
Vaginal	7/54 (13.2)	4/41 (9.7)	3/13 (23.1)	7/53 (13.2)	4/41 (9.7)	3/12 (25)	2/2 (100)
Pelvic site^c^	30 (18.3)	23 (18.2)	7 (18.4)	29 (17.9)	23 (18.7)	6 (16.7)	2 (50)

^a^The total of MRSA carriers were recruited at CUPS

^b^IDWS = Interdigital web spaces of hands

^c^Pelvic site = any of groin, perineal-perianal and vagina

Participants were most commonly colonised at multiple sites (Table 3). Among the 162 individuals colonised with MSSA, 25.3% were exclusively throat-colonised and 9.9% were exclusive nasal-colonised. Only one participant was exclusively colonised in the perineal-perianal region and one exclusively colonised on the upper back. There were no exclusive vaginal carriers. The proportion of those who had a positive throat culture who had a negative nasal culture was higher compared to the proportion of those who had a positive nasal culture who had a negative throat culture (41.5% vs. 31.7% p = 0.01). Among participants with negative nasal and throat cultures, the colonisation rate was less than 1.2% in non-pelvic sites and less than 3.7% in pelvic sites.

**Table 3:** Colonisation of a single site or multiple sites among the 162 MSSA carriers.

Culture site	MSSA	Multiple Sites	Single Site	Any site but throat	Any site but nose	Any site but throat and nose
	n (%)	n (%)	n (%)	n (%)	n (%)	n (%)
Nares	104 (64.2)	88 (54.3)	16 (9.9)	35 (20.1)	-	-
Throat	118 (72.8)	77 (47.5)	41 (25.3)	-	49 (30.2)	-
Upper back	22 (13.6)	21 (12.9)	1 (0.6)	6 (3.7)	9 (5.6)	2 (1.2)
IDWS^a^	33 (20.4)	33 (20.4)	0 (0)	5 (3.1)	7 (4.3)	1 (0.6)
Axillary	19 (11.7)	19 (11.7)	0 (0)	5 (3.1)	7 (4.3)	2 (1.2)
Groin	29 (17.9)	29 (17.9)	0 (0)	6 (3.7)	10 (6.2)	5 (3.1)
Perineal-perianal	29 (17.9)	28 (17.3)	1 (0.6)	8 (4.9)	12 (7.4)	4 (2.5)
Vaginal	7/53 (13.2)	7/53 (13.2)	0 (0)	5 (9.4)	2 (3.7)	2 (3.7)

^a^ IDWS = Interdigital web spaces of hands

### Risk factor analysis

Our hypothesis was based on an estimated prevalence of CA-MRSA of 8%, unexpectedly we found a low CA-MRSA colonisation rate of 1% and high MSSA carriage rate of 56.8%; thus, we assessed for risk factors associated with MSSA carriage instead.

As shown in Table 4, in the univariate analysis, age of 30 or less, part-time job, use of illicit drugs with strangers, having more than 6 heterosexual sexual partners in the last 6 months, practice of oral sex, trimming pubic hair with scissors, practice of any sport with mats (yoga, weight lifting or wrestling), and use of hot tubs, were all found to be associated with MSSA colonisation of any site. In the logistic regression, having a part-time job (OR 2.5, 95% CI 1.19–5.21), having more than 6 heterosexual sexual partners in the last 6 months (OR 5.9, 95% CI1.29–27.35) and trimming pubic hair (OR 1.8, 95% CI 1.01–3.51) remained significantly associated with MSSA carriage (Table 5). To analyse if these associations were driven by the STI clinic participants, we ran the logistic regression by site of recruitment (Table 6), finding that having a part-time job (OR 2.5, 95% CI 1.12–5.78) and trimming the pubic hair (OR 2.1, 95% CI 1.06–4.01) remained risk factors for colonisation among the STI clinic participants, having more than 6 heterosexual sexual partners in the previous 6 months only showed a trend (OR 3.9, p = 0.07)

**Table 4:** Univariate analysis of variables associated with MSSA colonisation.

Characteristic	MSSA positive				MSSA negative				p^f^
	STI clinic n = 126	CUPS n = 36	p^e^	Total n = 162	STI clinic n = 69	CUPS n = 54	p^e^	Total n = 123	
Age ≤30 years	67 (53.2)	6 (16.6)	**<0.01**	73 (45.1)	31 (44.9)	5 (9.2)	**<0.01**	36 (29.3)	**<0.01**
Part-time job	34 (26.9)	5 (13.8)	0.10	39 (24.1)	9 (13.0)	3 (5.5)	0.16	12 (9.7)	**<0.01**
Antibiotic use^a^	36 (28.6)	13 (36.1)	0.38	49 (30.6)	18 (26.1)	17 (31.5)	0.51	35 (28.5)	0.74
Self-reported infected wounds^a^	10 (7.9)	1 (2.78)	0.28	11 (6.8)	3 (5.6)	2 (2.9)	0.46	5 (4.1)	0.32
Drug use with strangers	0 (0)	8 (22.2)	0.12	8 (4.9)	0 (0)	0 (0)	-	0 (0)	**0.01**
Having any same-sex partners	35 (27.8)	2 (5.5)	<0.01	37 (22.8)	23 (33.3)	4 (7.4)	<0.01	27 (21.9)	0.76
Men having sex with men	29 (23.1)	0 (0)	<0.01	29 (17.9)	21 (30.4)	2 (3.7)	<0.01	23 (18.6)	0.86
>6 Heterosexual partners^a^	13 (10.3)	4 (11.1)	0.89	17 (10.5)	2 (2.9)	0 (0)	0.21	2 (1.6)	**<0.01**
Practice of oral Sex	121 (96.0)	20 (55.5)	**<0.01**	141 (87.0)	64 (92.7)	28 (51.8)	**<0.01**	92 (74.8)	**<0.01**
Removal of pubic hair	102 (80.9)	17 (47.2)	**<0.01**	119 (73.5)	57 (82.6)	22 (40.7)	**<0.01**	79 (64.2)	0.09
Trimming pubic hair with scissors	50 (39.7)	3 (0.8)	**<0.01**	53 (32.7)	18 (26.1)	4 (7.4)	**<0.01**	22 (17.8)	**<0.01**
Shaving pubic hair with razor	59 (46.8)	14 (38.8)	0.39	73 (45.1)	38 (55.1)	19 (35.2)	**0.02**	57 (46.3)	0.83
Other mode of hair removal^b^	17 (13.5)	0 (0)	**<0.01**	17 (10.5)	9 (13.0)	1 (1.8)	**0.02**	10 (8.1)	0.49
Plays hockey	13 (10.3)	1 (2.7)	0.15	14 (8.6)	4 (5.8)	0 (0)	0.07	4 (3.2)	0.06
Participants of wrestling	8 (6.3)	0 (0)	0.12	8 (4.9)	1 (1.4)	0 (0)	0.37	1 (0.8)	**0.05**
Practise weight lifting	42 (33.3)	1 (2.7)	**<0.01**	43 (26.5)	21 (30.4)	1 (1.8)	**<0.01**	22 (17.9)	0.08
Practise yoga	35 (27.7)	0 (0)	**<0.01**	35 (21.6)	16 (23.2)	0 (0)	**<0.01**	16 (13.0)	0.06
Any sport with mats^c^	65 (51.6)	1 (2.7)	**<0.01**	66 (40.7)	29 (42.0)	1 (1.8)	**<0.01**	30 (24.3)	**<0.01**
Frequent use of hot tubs^d^	37 (29.3)	4 (11.1)	**0.02**	41 (25.3)	16 (23.2)	4 (7.4)	**0.03**	20 (16.2)	0.07

^a^In the last 6 months

^b^Other = including waxing or laser removal of pubic hair

^c^Any sports with mats including wrestling, weight lifting and yoga

^d^At least once a week

^e^p value of difference of proportion of MSSA positive (or MSSA negative) among STI clinic vs CUPS clinic

^f^p value of difference of total MSSA positive vs MSSA negative from both clinics.

**Table 5:** Multivariate analysis of risk factors associated with MSSA colonisation at any site among 285 participants.

Risk factor	OR	p	95% Confidence intervals
Part-time job	2.4	0.01	1.19–5.21
>6 heterosexual sexual partners^a^	5.9	0.01	1.56–31.96
Trimming pubic hair	1.8	0.04	1.01–3.51

^a^In the last 6 months.

**Table 6:** Multivariate analysis of risk factors associated with MSSA colonisation at any site by recruiting sites.

Risk factor	STI clinic			CUPS		
	OR	p	95% CI	OR	p	95% CI
Part-time job	2.5	0.02	1.12–5.78	2.4	0.2	0.5–11.61
>6 heterosexual partners^a^	3.9	0.07	0.85–18.71	-^b^	-	-
Trimming pubic hair	2.1	0.03	1.06–4.01	0.9	0.9	0.15–5.27

^a^In the last 6 months.

^b^All those who have > 6 heterosexual partners were MSSA colonised.

We then looked for risk factors with respect to specific body site colonisation. Throat carriage was associated with practice of oral sex (OR 2.1, 95% CI 1.04–4.37), having more than 6 heterosexual sexual partners in the last 6 months (OR 1.8, 95% CI 1.05–3.09) and trimming the pubic hair (OR 2.7, 95% CI 1.54–4.77) (Table 7a). In contrast, nasal carriage was associated with trimming of pubic hair (OR 1.8, 95% CI 1.06–3.15), and practice of yoga (OR 2.1, 95% CI 1.17–4.05) (Table 7b).

**Table 7:** Multivariate analysis of risk factors associated with MSSA colonisation among 285 participants.

Risk factor	OR	p	95% CI
**A. MSSA throat colonisation**			
Practice of oral sex	2.1	0.03	1.04–4.37
>6 heterosexual sexual partners^a^	1.8	0.03	1.05–3.09
Trimming pubic hair	2.7	<0.01	1.54–4.77
**B. MSSA nasal colonisation**			
Trimming pubic hair	1.8	0.03	1.06–3.15
Practise yoga^b^	2.1	0.01	1.17–4.05

^a^In the last 6 months

^b^Due to the potential for confounding, the individual mat-associated sports were analysed individually.

### Molecular analysis

**Fig 2:**
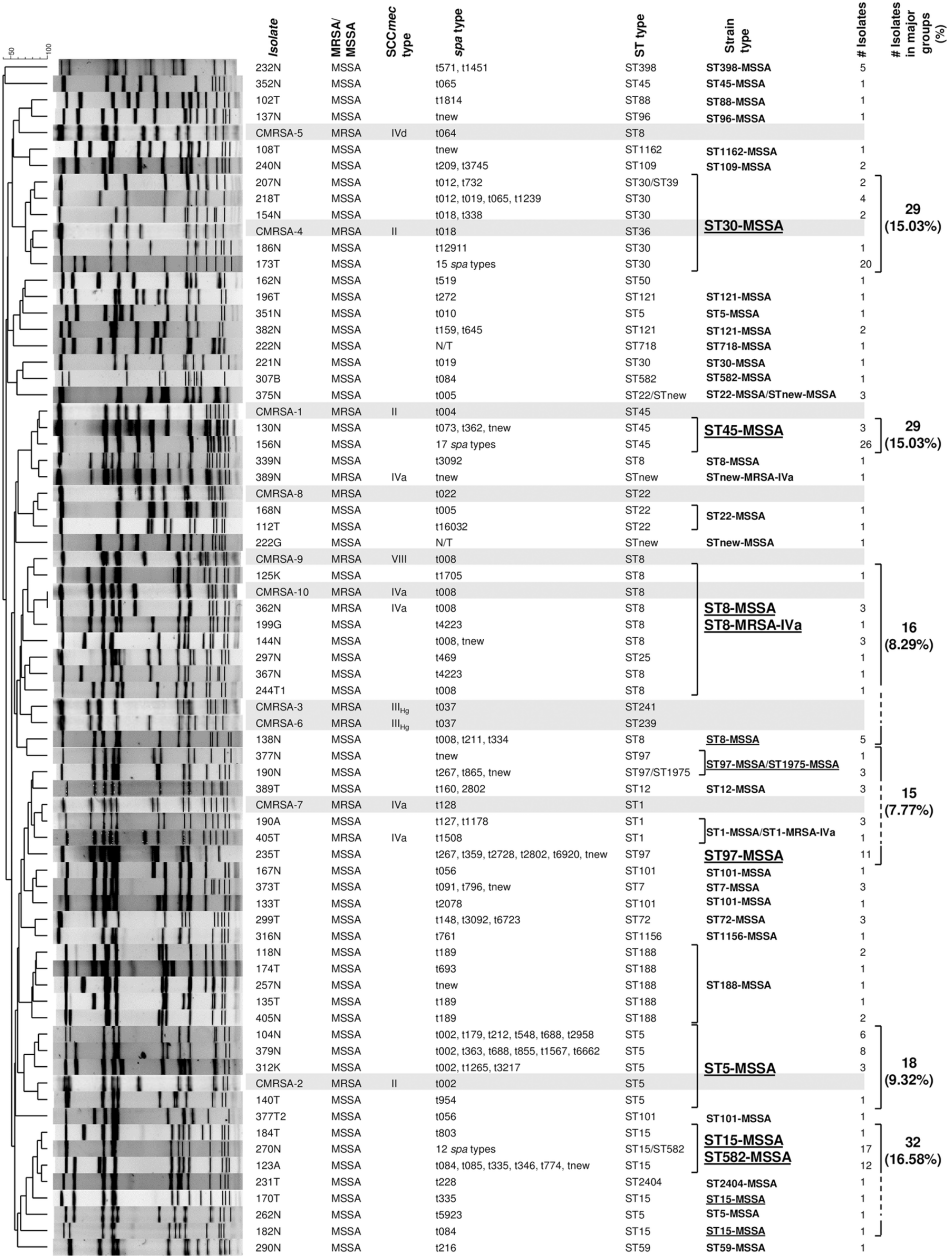
Molecular and genotypic characteristics of *Staphylococcus aureus* strains and isolates recovered from patients. Strains as described in the text, were identified using pulsed-field gel electrophoresis (PFGE) to identify major clonotypes. Isolates within each clonotype group were closely related, differing by only 2–3 bands. Molecular characterization identified 6 major types (underlined), these types were correlated with *spa* or MLST typing and are listed in the figure. All superscripts designations indicated either a new spa type or an unassigned type.

(Fig 2) A total of 193 isolates, including different strains from 164 participants, were analyzed. Molecular evaluation of the strains revealed highly diverse genotypes for MRSA and particularly MSSA grouped within 6 major clones. Three out of four MRSA colonised participants carried the USA300 strain determined by presence of SCC*mec* type IVa and PVL, spa type t008 and MLST type ST8. Of note, 12 MSSA isolates were spa type t008 and MLST type ST8 and showed more than 85% relatedness in the PFGE profile to the MRSA ST8-t008 (USA300-MRSA strain).

## Discussion

The results of this study indicate that the epidemiology of *Staphylococcus aureus* in this at-risk population is changing. We found a prevalence of SA carriage of 56.8%, with an high rate of MSSA carriage among participants attending a STI clinic (64.6%), compared to a similar study done in a marginalized urban population in Calgary in 2004 [23], where the colonisation rate was 42% and to community-based surveillance reports worldwide, where the rate of SA colonisation was 22–31.6%. [24,25] In contrast, we found a low prevalence of MRSA carriage of 1.4% compared to 7.4% found by Gilbert in Calgary in 2004. [23] Similar to our findings, others have reported a decline in MRSA in the general population and among persons infected with HIV. [26–28]

It has been proposed that the rates of MSSA colonisation in the community may reflect the intensity of environmental exposure [29] or complex network of people in the community with increased risk for SA acquisition. Interestingly our results exemplified this grade of exposure associated with certain risk factors. When assessing for specific body site of MSSA colonisation we found a preferential throat carriage of 72.8% compared to the nares (64.2%) which has been considered the traditional site of colonisation of SA with an approximate rate of colonisation of 30% in healthy individuals. [30,31] Our results showed that throat carriage was associated with certain sexual behaviours including having more than 6 heterosexual sexual partners in the last 6 months, practicing oral sex and trimming the pubic hair with scissors. These finding have a plausible biologic explanation, with more exposure through oral sex and multiple sexual partners suggesting a higher risk of throat colonisation. An exclusive throat colonisation rate in this study (25.3%) was higher than those reported previously in community-based studies including blood donors and in-hospital patients with rates of 16% and 12.4% respectively. [32]

Our findings support that certain risk behaviours are related to MSSA colonisation. The practice of oral sex has not been described as a risk factor for SA colonisation in the past. Miko and colleagues [9] did not find association with any specific self-reported number of sexual partners or sex practises among people attending a STI clinic. Similarly in a study of MRSA colonisation among HIV infected people [26], no specific behaviours were associated with carriage, but SA colonisation was higher among people with recent STIs suggesting risky sexual behaviour. Another risk factor found in our study was having a part-time job. Having a part-time job may represent a surrogate marker for a lower socioeconomic status, which has been recognized as a risk factor for MSSA/MRSA colonisation in prior studies. [33]

Anecdotally, removal of pubic hair was found to be common among sexually active individuals in our local population. Trimming of pubic hair instead of shaving, was associated with higher throat and nasal colonisation. This may reflect the intensity of manipulation of the area resulting in colonisation of the pubic area or MSSA hand inoculation and then self-inoculation to other body sites. MSSA carriage in pelvic sites was 18%, similar to 23% reported among patients attending a STI clinic in Baltimore. [9] A nasal and rectal only culture survey would have missed 25% of SA colonisation in our study, highlighting the value of multiple-site testing including throat, nasal and pelvic sites.

Additionally, among 3 novel sites assessed, the interdigital web spaces of the hands was the third most common site of SA carriage (21%), followed by the perineal-perianal area (18%) and the upper back (14%). Our study also found the practise of yoga vs any sports with mats, as a novel risk factor associated with MSSA colonisation. This association is biologically plausible, when people exercise with exposed upper back and has close contact with the mat while doing yoga. Prior studies suggest that skin contact is important for transmission of SA, including sharing of gym equipment [34] including mats [35], soaps [3] and towels. [36] Additionally, it is known that SA can be shed into the environment through desquamation of the skin, especially in patients with psoriasis and eczema. [37] This finding requires further investigation to confirm the presence of MSSA on mats.

Interestingly, we found 12 MSSA strains with high homology (>85%) to the ST8 t008 strains of MRSA (USA300 strain), raising the question of possible loss of methicillin resistance by complete or partial excision of the SCC*mec* element among the epidemic MRSA strain previously documented in this population [5], and may in part account for the decrease in the rates of MRSA colonisation. This mechanism has been recognized in MRSA nasal carriers with co-colonisation with MSSA that showed identical MLST and spa typing, and PCR amplification of *attB* insertion site confirmed SCC*mec* excision in vivo. [38] It has been shown that in the absence of selective pressure, mecA-negative or SCC*mec*-excised strains can arise *in vivo* secondary to spontaneous deletion of the methicillin resistant determinant. [39] The SCC*mec* deletion has also been associated with increased fitness in mixed culture competition studies [40–42] that may account for its increased adaptability and spread among people.

Our study has several limitations; we included participants from an urban inner-city clinic that provides dental and medical care and from an urban-core STI clinic. Thus, participants had significant baseline differences; however, they both represent populations at-risk for SA colonisation. The original study design was based on a sample size to assess risk factors of CA-MRSA assuming a prevalence of 8%, but instead we found a 7-fold higher rate of carriage of MSSA and only 1% of CA-MRSA. We therefore, performed a risk analysis for MSSA carriage with the original sample size, which was strong enough to detect associations. Due to the cross-sectional design of the study, colonisation status and risk factors were only assessed at one time point and these associations cannot be temporally determined. This study targeted a limited at-risk population in Calgary attending inner-city clinics and results may not be generalizable to other community populations.

Given the study design, volunteerism bias may have limited the participation of all patients at risk. The accuracy of the data may have been subject of underreporting bias due to lack of willingness to report sexual or drug practices, similarly self-reported comorbidities may not be accurate; we believe these limitations are not major concerns since the information collected in the questionnaires is similar to the profile of the population attending these clinics.

The current investigation has a number of strengths, including a large number of participants from a community-based setting, testing multiple body sites including 3 novel sites to minimize the risk of missing intermittent carriers, and the use of broth cultures, which have been shown to increase the yield of recovery of SA by 15–20%, compared to solid media. [13,43] Additionally, we were able to identify specific sexual behaviours associated with specific body sites of colonisation.

## Conclusions

Our study suggests a changing epidemiology of *Staphylococcus aureus* in this at-risk population attending inner-city clinics in Calgary, where we found a low prevalence of MRSA colonisation and high prevalence of MSSA compared to 22–32% prevalence reported in Canada and worldwide. The throat represents an important reservoir of MSSA in the community and may reflect the degree of exposure through sexual behaviour. Nasal carriage of MSSA was strongly associated with practice of yoga, having important epidemiological consequences for transmission. Close strain relatedness of MSSA and USA300-MRSA isolates suggests gain or loss of SCC*mec* element that requires further study.

## Supporting information

S1 File Study questionnaire. (PDF)
