# Supplementary material for: The epidemiology of Staphylococcus aureus carriage in patients attending inner city sexually transmitted infections and community clinics in Calgary, Canada
Source: PLoS One. 2017 May 25;12(5):e0178557. doi: 10.1371/journal.pone.0178557 (PMC5444836; doi:10.1371/journal.pone.0178557)
Supplement: S1 File — (PDF) [file pone.0178557.s001.pdf]

Date of interview

[ ]
Day/Month/Year

AFFIX STUDY LABEL HERE

1 **What year were you born?** Year= ______ Don't know [ ] Refused [ ]

2 **Sex:** Female [ ] Male [ ] Don't know [ ] Refused [ ]

3 **What ethnic group do you most identify with?** Don't know [ ] Refused [ ]
Caucasian [ ] Asian [ ] Aboriginal [ ] Other [ ] Don't know [ ]
If aboriginal Metis [ ] Inuit [ ] First Nation [ ]
If First Nation Status [ ] Non-status [ ]

4 **Do you have a job?** No Job [ ] Don't know [ ] Refused [ ]
Full time [ ] Part-time [ ] Casual or Day Labour [ ]

5 **What are some other ways you get or make money?** Don't know [ ] Refused [ ]
AISH [ ] EI [ ] Welfare [ ] Other way [ ] ______

6 **Have you lived in Calgary for the last 2y?** Yes [ ] No (go to#8) [ ] Refused [ ]

7 **How long have you lived in Calgary** < 1 month [ ] 1-3 months [ ] 4 month-1y [ ] >1 y [ ]

8 **In the last 6 months what kinds of places have you lived in?** (check all that apply)

**At all**		**Living now**
[ ]	Own appartment /house	[ ]
[ ]	Friend's place	[ ]
[ ]	Hotel or motel room	[ ]
[ ]	Roaming or boarding house	[ ]
[ ]	Transition or half way house	[ ]
[ ]	On the street	[ ]
[ ]	Jail	[ ]
[ ]	Shelter or detox house	[ ]
[ ]	Don't know	[ ]
[ ]	Refused	[ ]

9 **If in jail**
How long was your longest stay in the past 6m?

[ ] <1week
[ ] >1w but <1m
[ ] >1month
[ ] Don't know
[ ] Refused

10 **If you have lived in a shelter or detox centre which ones? (check all that apply)**

Alpha house [ ] Mustard seed [ ] Don't know [ ]
Renfrew Recovery [ ] Drop-in Ctr [ ] Refused [ ]
Simon House [ ] Centre of Hope [ ]
Aventa [ ] The Warehouse [ ]
Servants Anonymous Soc [ ] The Booth [ ]
Fresh start [ ] Sunalta/2035 [ ]
Sunrise residence [ ] Centre 16 [ ]
Other ______ Electrical Station [ ]

11 **In the place you live in now, how many people do you live with?** ______ Don't know [ ] Refused [ ]

12 **Which neighbourhood do you spend most of your time in? (not sleeping or for job)**

Bowness [ ] Downtown [ ] Other NW [ ] Other SE [ ]
Forest Lawn [ ] Inglewood [ ] Other NE [ ] Don't know [ ]
Victoria Park [ ] Albert Park [ ] Other SW [ ] Refused [ ]

The next set of questions are about drugs and alcohol. We are asking everyone in this study the same questions.

13 **How often do you drink alcohol?**

Almost everyday [ ] Several times a month [ ] Don't know [ ]
Several times a week [ ] Rarely or never [ ] Refused [ ]

14 **In the past <u>6 months</u>, have you used any recreational drugs?** Yes ☐ No (go#22) ☐ Refused ☐

15 **In the past <u>6 months</u>, which of the following drugs have you used?** (check all that apply)

16 **Which drug do you use most often?** (check one)

	inject	smoke	other	16
Cocaine	☐	☐	☐	☐
Crack cocaine	☐	☐	☐	☐
Morphine other narcotics	☐	☐	☐	☐
Heroin	☐	☐	☐	☐
Cocaine+morphine	☐	☐	☐	☐
Cocaine+heroin	☐	☐	☐	☐
Crystal meth	☐	☐	☐	☐
Marijuana	☐	☐	☐	☐
Other drugs	☐	☐	☐	☐
Don't use drugs (go to # 20)	☐	☐	☐	☐
Don't know	☐	☐	☐	☐
Refused	☐	☐	☐	☐

17 **For the drug you use most often, how often do you use this drug?**

- >5 times a day ☐
- <5 times a day but > once ☐
- Once a day ☐
- <5 days a week ☐
- Once a week ☐
- Don't know ☐
- Refused ☐

18 **In the last <u>6 months</u> did you go on runs or binges when you used drugs more than usual?**

- Yes ☐
- No (go to # 21) ☐
- Don't' know ☐
- Refused ☐

19 **How many times did you binge?**

_________ times per month

_________ times in 6 months

20 **How long did they last?**

- 1 day ☐
- 2-5 days ☐
- >5 days ☐
- Don't know ☐
- Refused ☐

21 **In the past <u>6 months,</u> who have you used drugs with?** (check all that aply)

- Regular sex partner(s) ☐
- Casual sex partner (s) ☐
- With a date or a STW ☐
- Close friends or family ☐
- People you don't know well ☐
- People tou don't know at all ☐
- Always use alone ☐
- Don't know ☐
- Refused ☐

22 **In the past <u>6 months</u>, have you lived with someone who uses drugs?**

- Yes ☐
- No (go to # 24) ☐
- Don't know ☐
- Refused ☐

23 **Which drugs did this person use?** (check all that apply)

- Cocaine ☐
- Crack cocaine ☐
- Morphine/other narcotics ☐
- Heroin ☐
- Crystal meth ☐
- Other drugs ☐
- Don't know ☐

The next set of questions are related to your life in Calgary

24 **In the last <u>6 months</u> how often have you eaten at any community dinners or soup kitchens?**

- Never ☐
- Once a week ☐
- 2-4 times/week ☐
- >5times/week ☐
- Daily ☐
- Don't know ☐
- Refused ☐

25 **Which ones?** (check all that apply)

- Mustard seed ☐
- Alpha house ☐
- Centre of hope ☐
- Drop-in Ctr ☐
- Inn from the cold ☐
- St Mary's ☐
- The Booth ☐
- CUPS/FRC ☐
- Other: _________
- Refused ☐

The next set of questions are about how your health has been recently

26 **In the last <u>2 years</u>, have you been admitted to the hospital?**

- Yes ☐
- No ☐

27 **How many times in the last:**

6 months _________

1 year _________

2 years _________

28 ***For the last hospitalization***

a **How long ago were you in hospital?**

- <1 month ☐
- 1-6 months ☐
- 7-12 mos ☐
- >1 year ☐
- Don't know ☐
- Refused ☐

b **How many days were you in hospital for?**

- <24 hours ☐
- 1-3 days ☐
- 4-7 days ☐
- >week ☐
- Don't know ☐
- Refused ☐

c **Why were you admitted to hospital?**
(check one)

- Surgery (emergency or planned) ☐
- Medical reason ☐
- Psychiaty ☐
- Other reason ☐
- Don't know ☐
- Refused ☐

d **Did any of the following things happened while you were in hospital?**

- Stay in ICU ☐
- Nasogastric tube ☐
- Urinary catheter ☐
- Dialysis ☐
- IV catheter ☐
- Endotracheal tube ☐
- Don't know ☐
- Refused ☐

29 **Have you taken or received any antibiotics in:**
<u>Last 6 months?</u> Yes ☐ No ☐

30 **In the last 2 weeks**

	Have you had any pimples or boils	Have you had any infected wound or cut
Face	☐	☐
Back	☐	☐
Upper tighs	☐	☐
Genitalia	☐	☐
Injection site	☐	☐
Other	☐	☐

31 **In the last 6 months**

	Have you had any pimples or boils	Have you had any infected wound or cut
Face	☐	☐
Back	☐	☐
Upper tighs	☐	☐
Genitalia	☐	☐
Injection site	☐	☐
Other	☐	☐

	Yes	No	Refused
32 **Did you get a doctor or nurse to look at these infections?**	☐	☐	☐
33 **Did you squeeze, pop or cut them on your own?**	☐	☐	☐
34 **Did someone else squeeze, pop or cut them for you?**	☐	☐	☐
35 **Did you take antibiotics for them?**	☐	☐	☐
36 **In the past <u>6 months</u> have you lived with or been in close physical contact with anyone with a skin infection?**	☐	☐	☐

37 **Have you ever been diagnosed with:**
(check all that apply)

- Hepatitis C ☐
- Hepatitis B ☐
- HIV ☐ CD4 count ____________ date ____________
- Diabetes ☐
- Skin condition (psoriasis, acne eczema) ☐
- A heart valve infection endocarditis ☐
- A bone infection (osteomyelitis) ☐
- A joint infection (septic arthritis) ☐
- Lung condition (chronic) ☐
- A kidney disease (chronic) ☐
- A liver condition (no hepatitis B/C) ☐
- Cancer ☐
- Don't know ☐
- Refused ☐

The next section deals with sexual practices

38 **In the last <u>6 months</u>:**

a **How many different partners of the same gender have you have sex with:**

b **How many partners of the opposite gender have you had sex with:**

a		b
☐	None	☐
☐	1	☐
☐	2-5	☐
☐	6-10	☐
☐	11-20	☐
☐	>20	☐

39 **Do you use condoms?**

Never ☐ Sometimes ☐ Almost always ☐ Always ☐

If sexual partner of same gender. In the last <u>6 months</u>...

	Yes	No
40 **Have you practice receptive oral sex**	☐	☐
41 **Have you practiced receptive anal sex (bottom)**	☐	☐
42 **Have you practiced insertive anal sex (top)**	☐	☐

43 **What are your usual sexual practices (check all that apply:**

- Insertive anal sex ☐
- Receptive anal sex ☐
- Oral sex ☐
- Mutual masturbation ☐

This sectiondeals with body grooming patterns and sport activities

44 **Do you have any peircings?**

- Yes ☐
- No ☐
- Refused ☐

45 **Where are your peircings?** (check all that apply)

- Belly button ☐
- Penis/labia ☐
- Testicles ☐
- Ear lobe ☐
- Perineum ☐
- Nose ☐
- Tonge ☐
- Nipple ☐

46 **Do you remove your pubic hair?**

- Yes ☐ Shave ☐ Wax ☐ Trim ☐
- No ☐

47 **Do you participate regularly in any of the following sports?** (check all that apply)

- Football ☐
- Hockey ☐
- Wrestling ☐
- Soccer ☐
- Swimming ☐
- Weightlifting ☐

48 **In what capacity do you participate?**

- Team player ☐
- Coach ☐
- I do not play ☐

49 **Do you attend the gym?**

- Yes ☐
- No ☐
- Refused ☐

50 **What activities you indertake in the gym?**

- Weights ☐
- Cardio ☐
- Yoga ☐
- Pilates ☐
- Swimming ☐
- Hot tub ☐
- Steam room ☐
- Sauna ☐
- Shower at the gym ☐

Can you show me ALL the skin infections that you have right now? I would like to have at look at them and if possible take a swab.

A skin infection must meet the following criteria:

1 A wound with purulent drainage; or pustules, vesicles or boils with or without purulent drainage OR

2 Two of the following at an affected site: localized pain or tenderness, swelling, readness or heat.

Swabs should be collected in the infection is open or draining, or if the infection is a pustule or other superficial contained collection of pus. Deeper or non-pustular lesions such as boils or abscesses which are not draining should not be swabbed.

If the participant identifies more than two skin infections: priority should first be given to infections that can be swabbed as described above, and the to infections that in your clinical judgment appear to be most severe. Please record information on up to two infections even if they cannot be swabbed.

1 **Infection site # 1** **Localization**____________________

2 **Type of infection:** (check one)

Infected wound, cut, laceration, injury ☐ Blister ☐ Other ☐ ________

Pustule (folliculitis) ☐ Cellulitis ☐

Boil (furuncule, cabuncle) ☐ Impetigo ☐

3 **Is this infection site:** (check one) Swabbed Labeled Infection1

Open or draining purulent-material (should swab) ☐ ☐ ☐

Open or draining non-purulent material (should swab) ☐ ☐ ☐

Not open or draining, but is a superficial collection of pus or pustule (swab) ☐ ☐ ☐

Not open or draining, with no superficial collection of pus (don't swab)

4 **Was the infection an injection site that became infected?**

Yes ☐ No ☐ Don't know ☐

5 **What other characterisitics does this infections have?** (check all that apply)

Redness ☐ Swelling ☐ None of the above ____

Localized pain or tenderness ☐ Heat ☐

6 **Was the participant referred on to medical care because of this infection?**

Yes ☐ No ☐ Don't know ☐

1 **Infection site #2** **Localization**____________________

2 **Type of infection:** (check one)

Infected wound, cut, laceration, injury ☐ Blister ☐ Other ☐ ________

Pustule (folliculitis) ☐ Cellulitis ☐

Boil (furuncule, cabuncle) ☐ Impetigo ☐

3 **Is this infection site:** (check one) **Swabbed** **Labeled Infection2**

Open or draining purulent-material (should swab) ☐ ☐ ☐

Open or draining non-purulent material (should swab) ☐ ☐ ☐

Not open or draining, but is a superficial collection of pus or pustule (swab) ☐ ☐ ☐

Not open or draining, with no superficial collection of pus (don't swab)

4 **Was the infection an injection site that became infected?**

Yes ☐ No ☐ Don't know ☐

5 **What other characterisitics does this infections have?** (check all that apply)

Redness ☐ Swelling ☐ None of the above ____

Localized pain or tenderness ☐ Heat ☐

6 **Was the participant referred on to medical care because of this infection?**

Yes ☐ No ☐ Don't know ☐
